# Editorial: A Collection of Systematic Reviews or Meta-Analyses on the Effects of Behavioral and Psychosocial Interventions for Psychological Well-Being

**DOI:** 10.3389/fpsyg.2022.850608

**Published:** 2022-03-24

**Authors:** Ai Bo, Iris Chi, Fang Fu, Zhenggang Bai

**Affiliations:** ^1^Department of Social Work, Helen Bader School of Social Welfare, University of Wisconsin–Milwaukee, Milwaukee, WI, United States; ^2^Suzanne Dworak-Peck School of Social Work, University of Southern California, Los Angeles, CA, United States; ^3^Department of Social Work, School of Social Development and Public Policy, Fudan University, Shanghai, China; ^4^Evidence-Based Research Center of Social Science and Health, School of Public Affairs, Nanjing University of Science and Technology, Nanjing, China

**Keywords:** systematic review, meta-analysis, randomized controlled trial (RCT), evidence-based practice (EBP), psychosocial intervention

Intervention research evaluating the efficacy or effectiveness of behavioral and psychosocial interventions on psychological well-being and behavioral health outcomes across the life span has grown tremendously in recent decades. Following the principles of evidence-based practice, mental health and health care professionals draw on the best available research evidence along with their clinical expertise and client preferences to inform their clinical decision-making when selecting the most effective and appropriate intervention programs for their clients (American Psychological Association, 2006). However, given the recent dramatic increase in the number of scientific publications in this area and the often inconsistent conclusions they yield, it is difficult for research consumers to identify the best available evidence. Systematic reviews and meta-analyses of randomized controlled trials (RCTs) are considered to provide the strongest evidence because they (a) employ a structured approach to identifying and evaluating empirical evidence, (b) appraise the evidence for internal validity and clinical usefulness, and (c) have greater statistical power and precision for estimating treatment effects than do individual studies (Mulrow, 1994; Evans, 2003).

We initiated this Research Topic in recognition of the extant published systematic reviews with or without meta-analyses on the efficacy or effectiveness of behavioral and psychosocial interventions aimed at treating behavioral problems or enhancing the psychological well-being of participants across the life span. We identified a total of nine systematic reviews in this Research Topic, each of which synthesizes a body of empirical evidence on the efficacy or effectiveness of various behavioral and psychosocial interventions for different populations, including children, youth, adults, and older adults. The systematic reviews also identify knowledge gaps and offer recommendations for ongoing intervention research.

## Interventions for Children and Youth

Three systematic reviews focused on behavioral and psychosocial intervention programs for children and youth.

Li et al. synthesized recent empirical evidence drawn from 37 studies (36 RCTs and one quasi-experimental study published from 2013 to 2020) of the Triple P (Positive Parenting Program) on social, emotional, and behavioral outcomes among many different child populations, ranging from the general population to those at risk for behavioral problems. The meta-analysis found that Triple P yielded statistically significant increases in children's social competence and reductions in their emotional and behavioral problems. The review also found supportive evidence that Triple P reduced negative parenting styles, conflicts over parenting, parents' mental distress, and parent-child conflict, while improving parental self-efficacy. The review also identified a few intervention and participant characteristics that moderated the intervention's effects on child and parent outcomes.

Chan et al.'s review synthesized the effects of experiential and non-experiential learning programs on prosocial behavior, empathy, and subjective wellbeing among children and youth aged 8–25 years based on 20 RCTs. The meta-analysis found that experiential learning programs yielded statistically significant effect sizes on empathy and on subjective wellbeing, based on four studies and five studies, respectively. Statistically significant effects were not found for non-experiential learning programs for any of the three outcomes.

Aithal et al. reviewed nine studies with various research designs that evaluated the initial evidence of dance movement psychotherapy interventions on the wellbeing of children with autism spectrum disorders and found that these interventions yielded promising improvements in various social and communication skills. However, the review indicated that strong empirical evidence for the efficacy of dance movement psychotherapy is still lacking.

## Interventions for Adults

Four systematic reviews addressed behavioral and psychosocial interventions for adults.

Wang et al. synthesized five RCTs of dance-based interventions on depression and anxiety among persons with mild cognitive impairment and dementia. The review found a statistically significant average effect size of danced-based programs on reducing depressive symptoms and insufficient evidence of an effect on anxiety symptoms.

Velana et al. reviewed 27 relevant studies (including RCTs and quasi-experimental studies published from 2000 to 2020) of the individual interventions on stress management among nurses. The review suggested that technology-delivered interventions with relaxation and stress management interventions comprising cognitive-behavioral components might effectively decrease stress among nurses and improve their well-being.

Yang et al. synthesized five RCTs to explore the effects of art therapy (i.e., music therapy and painting therapy) on depression, anxiety, blood glucose, and glycated hemoglobin in patients with diabetes mellitus (DM). The results provided supportive evidence of the beneficial effects of art therapy on depression and blood glucose in patients with DM.

Zhou et al. synthesized six RCTs to examine the effect of internet-based interventions (IBI) on veterans' post-traumatic stress disorder (PTSD) symptoms. The review found supportive evidence of IBI's ability to reduce overall PTSD outcomes. Subgroup analysis showed that IBI-based cognitive behavioral therapy with peer support had a beneficial effect on PTSD outcomes among veterans. Subgroup analyses were also conducted based on outcome measures and comorbidity of participants.

## Interventions for Older Adults

Two systematic reviews and meta-analyses reviewed behavioral and psychosocial interventions for older adults.

Shen et al. synthesized six studies (i.e., four RCTs and two quasi-experimental studies) with 51 effect size estimates, and reported an overall positive and statistically significant treatment effect of psychosocial interventions for elder abuse-related outcomes. Interventions that used a family-based model, combined education and supportive services, and targeted both caregivers and elders yielded a significant effect size, suggesting such features should be considered for inclusion in elder abuse intervention design.

Jin et al. synthesized six RCTs to examine the effectiveness of technology-based interventions for reducing loneliness in older adults. Their meta-analysis did not find supportive evidence of the efficacy of the technology-based interventions in loneliness reduction.

## Conclusion and Future Directions

Interventions reviewed in this Research Topic are all multicomponent psychosocial interventions that are either (a) broad-based programs addressing multiple positive developmental outcomes among a wide range of populations of interest or (b) more targeted programs designed to reduce specific problems among at-risk populations. For either type of program, the intervention effects may vary based on intervention and participant characteristics. Although some of the included reviews tried to shed light on the moderators of the intervention effects using subgroup analysis, subgroup analysis often does not have sufficient statistical power to detect significant effects due to the limited number of included studies and effect sizes. Further, intervention mechanisms remain largely unknown because such information is often unrecorded by the original studies. Therefore, the authors of these reviews called for more high-quality RCTs with long-term follow-ups to further understand the effects and mechanisms of the interventions. Nonetheless, these reviews are extremely beneficial because they synthesized the most recent empirical evidence and identified knowledge gaps to be studied by future intervention research.

## Author Contributions

All the authors contributed to drafting the manuscript. AB and IC also contributed to revising the manuscript. All authors contributed to the article and approved the submitted version.

## Conflict of Interest

The authors declare that the research was conducted in the absence of any commercial or financial relationships that could be construed as a potential conflict of interest.

## Publisher's Note

All claims expressed in this article are solely those of the authors and do not necessarily represent those of their affiliated organizations, or those of the publisher, the editors and the reviewers. Any product that may be evaluated in this article, or claim that may be made by its manufacturer, is not guaranteed or endorsed by the publisher.
